# Commentary on the Regulation of Viral Proteins in Autophagy Process

**DOI:** 10.1155/2014/962915

**Published:** 2014-03-10

**Authors:** Ching-Yuan Cheng, Pei-I Chi, Hung-Jen Liu

**Affiliations:** ^1^Institute of Molecular Biology, National Chung Hsing University, Taichung 402, Taiwan; ^2^Agricultural Biotechnology Center, National Chung Hsing University, Taichung 402, Taiwan; ^3^Rong Hsing Research Center for Translational Medicine, National Chung Hsing University, Taichung 402, Taiwan

## Abstract

The ability to subvert intracellular antiviral defenses is necessary for virus to survive as its replication occurs only in the host cells. Viruses have to modulate cellular processes and antiviral mechanisms to their own advantage during the entire virus life cycle. Autophagy plays important roles in cell regulation. Its function is not only to catabolize aggregate proteins and damaged organelles for recycling but also to serve as innate immunity to remove intracellular pathogenic elements such as viruses. Nevertheless, some viruses have evolved to negatively regulate autophagy by inhibiting its formation. Even more, some viruses have employed autophagy to benefit their replication. To date, there are more and more growing evidences uncovering the functions of many viral proteins to regulate autophagy through different cellular pathways. In this review, we will discuss the relationship between viruses and autophagy and summarize the current knowledge on the functions of viral proteins contributing to affect autophagy process.

## 1. Introduction

Autophagy is an intracellular degradative process including macroautophagy, microautophagy, and chaperone-mediated autophagy (CMA) [1]. In this review, we focus on the regulation of viral proteins in microautophagy (referred here as autophagy) process. Autophagy can take place in a manner of selected or nonselected catabolic process via the lysosomal pathway as compared to ubiquitin-proteasome degradation. Under starvation stress, autophagy provides the energy source for cell survival by degradation of the unwanted substance. Besides, autophagy is responsible for removing the damaged organelles caused by reactive oxygen species (ROS) [2]. Impaired autophagy had been found closely related to many pathological conditions, such as neurological disorders, aging, diabetes, and cancer [3]. Autophagy is also involved in the innate and adaptive immunity system to resist pathogen infection by viruses and bacteria [4, 5].

Many signaling pathways are involved in activation of autophagy. The mammalian target of rapamycin (mTOR) is one of the main negative mediators of autophagy. While the upstream negative regulators of mTOR such as phosphatase and tensin deleted on chromosome 10 (PTEN), AMP-activated protein kinase (AMPK), p53, eukaryotic translation initiation factor 2*α* (eIF2*α*), and c-jun-N-terminal kinase (JNK1) in turn function as activators of autophagy [6]. When mTOR is repressed, the interaction between mTOR and ULK1 (Atg1) become weak, therefore, resulting in hypophosphorylation of ULK1 as well as Atg13. The activated hypophosphorylated ULK1 is then associated with Atg13 and Atg17 to form a complex that promotes the initial stage of autophagy [7]. During this initiation step of autophagy, the Beclin 1 formed the complex with Vps34 and Atg14 that was essential for phagophore formation [8]. Members of the Bcl-2 family acted as inhibitors in this stage [9]. In the second step, elongation of the isolated membrane triggered the formation of double-layer phospholipid membrane that can engulf organelles. Activations of Atg7 and Atg10 catalyzed the formation of Atg12-Atg5-Atg16 complex [10]. On the other hand, the C-terminal arginine 117 residue of newly synthesized Atg8 is initially cleaved by the Atg4 in order to expose a glycine residue [11]. The Gly-terminal residue of Atg8 is then bound to the active cysteine 507 of Atg7 and then activated by Atg7 (an E1-like enzyme). The activated Atg8 is then transferred to another E2-like enzyme (Atg3). Finally, Atg8 is conjugated to the phosphatidylethanolamine (PE) by an amide bond between the C-terminal glycine of Atg8 and the amino group of PE [12]. Both of these two conjugation systems were important in promoting the elongated membrane curvature, thereby, resulting in the autophagosome formation. The third step of autophagy was the docking and fusion of autophagosome with lysosome through the assistances of LAMP2 and Rab7 proteins [13]. The inner membrane of autophagosome and organelles that were engulfed earlier were broken down into small molecules by lysosome enzymes such as cathepsins B, D, and L [3]. Finally, those small molecules were exported to cytoplasm and recycled as new sources for further uses.

Recently, growing numbers of studies have discovered that autophagy seems to involve in life cycle of some viruses. It is not surprised that xenophagy, a form of autophagy, is the cellular intrinsic immune system against viruses. Therefore, the suppression in autophagy provides virus with a way to overcome the cellular defense mechanisms. Vesicular stomatitis virus (VSV) inhibits cellular autophagy to facilitate viral replication in infected cells [14]. The elevated expression of Beclin 1 resulted in reduction of virus replication of both herpes simplex virus type 1 (HSV-1) and sindbis virus [15, 16]. Human cytomegalovirus (HCMV) and simian immunodeficiency virus type 1 (SIV 1) both had been reported to inhibit autophagy through different strategies [17–19]. Even though the autophagy is thought to be an intracellular antiviral system, however, numbers of viruses have been found to activate autophagy instead of repression. Previous studies have indicated that infections of Epstein-Barr virus (EBV) and varicella zoster virus (VZV) caused induction of autophagy [20, 21]. Furthermore, avian reoviruses (ARVs), coxsackievirus B3 (CVB3), dengue virus, hepatitis C virus (HCV), and influenza A virus (IAV) not only induced autophagy but also upregulated autophagy to enhance virus replication [22–26]. Interestingly, human immunodeficiency virus type 1 (HIV-1) suppresses the late stages of autophagy [27]; however, it has been reported that autophagy-related genes appear to facilitate HIV infection [28]. The different stages of autophagy might play different roles in virus life cycles. Formation of autophagosomes has been proposed to offer the platform for viral replication and viral assembly, like HIV-1 [18, 29]. Degradation of the lipids in the late stage of autophagy can provide the energy source for viral replication as dengue virus (DENV) [30]. Some viruses, like CVB3, trigger incomplete autophagy flux for their own benefits [3]. Furthermore, it has been reported that ARV triggers the fusion of autophagosome with lysosome into autolysosome without completing the autophagy flux [22]. How the virus regulates this incomplete autophagy process and what kinds of advantage this process brings to viruses still remain unclear.

## 2. The Contributions of Viral Proteins in Regulating Signaling Pathways of Autophagy

Many viral proteins have been demonstrated to play important roles in autophagy regulation either by interacting with autophagy-related proteins or by modulating the signaling pathways that affect the autophagy. Here we will focus on the regulations of Beclin 1/Vps34 complex, PKR/eIF2*α* signaling pathway, the autophagy pathways, mTOR signaling pathway, and cytoprotective signaling pathways and briefly summarize the roles of these well-characterized viral proteins in regulation of autophagy, as shown in Table 1.

### 2.1. Regulation of Beclin 1/Vps34 Complex

#### 2.1.1. The Bcl-2-Like Viral Protein

HSV-1 is a 152 kb double-stranded DNA virus, belonging to the *α*-herpesvirus family. HSV-1 negatively regulates autophagy during infection [31]. The *γ*_1_34.5 gene of HSV-1 encodes ICP34.5 protein, consisting of 263 amino acids. This protein has three domains including an N-terminal region, a linker region of Ala-Thr-Pro repeats, and a C-terminal region [32, 33]. It regulates cellular autophagy via two distinctive mechanisms. The N-terminal region of ICP34.5 possesses a Beclin 1 binding domain, which mimics the inhibitory effect of the cellular Bcl-2 protein, thereby, preventing Beclin 1 to bind with the PI3KC3- (phosphoinositide 3-kinase class III-) UVRAG (UV irradiation resistance-associated gene) complex. Beclin 1 is a critical component of PI3KC3 complex that triggers the initiation of autophagy, and its function is suppressed through interaction of Bcl-2 with the BH3 domain of Beclin 1 [34]. The HCMV protein TRS1 and the viral homologues of Bcl-2 of Kaposi's sarcoma herpesvirus (KSHV) and murine gamma herpesvirus 68 (*γ*HV-68) have been also shown to inhibit the formation of autophagosomes through their binding to Beclin 1 [9, 35, 36].

#### 2.1.2. The Hepatitis B Virus X Protein (HBx)

HBV is a double-strand DNA virus, a member of the hepadnavirus family. Its DNA genome contains four genes named S, C, P, and X, respectively. The HBx protein encoded by the X gene serves as a multifunctional regulatory protein since it is involved in regulating cell cycle, signaling transduction, and cellular apoptotic pathways [37]. HBV can enhance autophagic flux in cell culture and transgenic mouse liver and during natural infection. Enhancement of autophagy caused by HBV is now believed by the contribution of HBx [38]. In accordance with the results of coimmunoprecipitation assays performed by Sir et al., it was discovered that HBx can directly bind to Vps34, the catalytic subunit of PI3KC3, to promote the activity of PI3KC3. The HBx-induced autophagy benefits HBV DNA replication rather than RNA transcription. Furthermore, autolysosomes were not involved in HBV replication, since treatment of cells with bafilomycin to suppress formation of autolysosomes showed no effects on HBV DNA replication [38].

### 2.2. Regulation of PKR/eIF2*α* Signaling Pathway

#### 2.2.1. The Regulators of Protein Phosphatase 1 (PP1)

In addition to inhibition of autophagy by binding to Beclin 1, ICP34.5 can also regulate autophagy through regulation of eIF2*α*. eIF2*α* plays a central role in the maintenance of mRNA translation. Phosphorylation of eIF2*α* at Ser51 shuts off protein translation and stimulates autophagy [39]; therefore, eIF2*α* can be regarded as a modulator of autophagy. The C-terminal region of ICP34.5 contains a consensus binding motif (R/KVXF) for PP1 followed by an Ala-Arg-rich motif, that is, highly homologous to the C-terminal region of mammalian growth arrest and DNA damage protein 34 (GADD34) [40–42]. More recently, the amino acids residues 233–248 in the ICP34.5 (homology region of GADD34) were verified as a binding site of eIF2*α*. It has been also demonstrated that the binding between ICP34.5 and eIF2*α* is crucial for specific dephosphorylation of eIF2*α* by PP1, thus facilitating the initiation of protein translation and, as a result, suppressing autophagy [43]. The African swine fever virus (ASFV) DP71L protein and the pseudorabies virus (PRV) IE180 protein act similarly to ICP34.5 to abolish phosphorylation of eIF2*α* by regulating PP1 [44, 45].

#### 2.2.2. Us11

Us11, a late *γ*2 gene product, is a viral protein abundantly produced in the late stage of HSV-1 life cycle. Like ICP34.5, Us11 is involved in inhibition of eIF2*α* phosphorylation. Earlier studies have shown that Us11-null viruses grow normally* in vitro* and are slightly attenuated* in vivo*, suggesting that the function of Us11 may be compensated by the extant ICP34.5 [46, 47]. Nevertheless, Us11 acts in concert with ICP34.5 to inhibit autophagy through a mechanism totally different from that in which ICP34.5 is involved. Us11 blocks the kinase activity of protein kinase R (PKR) via directly binding to either PKR or upstream activators of PKR. Since eIF2*α* is the substrate of PKR, the PKR binding domain located between amino acids residues 91–121 close to the C-terminus of Us11 contributes to physical association with PKR, resulting in a reduction of eIF2*α* phosphorylation [48]. A previous investigation demonstrated that the truncated Us11 mutants without its N-terminus failed to inhibit poly- (I·C-) induced autophagy in HeLa cells stably expressing GFP-LC3, indicating that the N-terminal region of Us11 is critical for inhibition of autophagy [49].

It has been well-known that PKR is transcriptionally induced by interferon and activated by double-stranded RNA (dsRNA). The C-terminus of Us11 contains an Arg/Pro-rich RNA binding domain (amino acids residues 91–152), which can bind to dsRNA to dampen the stimulation of PKR [50]. In addition, Us11 also can interact with activators of interferon, including retinoic acid-inducible gene I (RIG-I), melanoma-associated differentiation gene 5 (MDA-5), and the protein activator of the interferon-induced protein kinase (PACT), to inhibit the production of interferon through its C-terminal RNA binding domain [51, 52].

#### 2.2.3. Murine Cytomegalovirus (MCMV) m142 and m143 Proteins

MCMV is a DNA virus in the Herpesviridae family. MCMV gene expression and/or viral replication can cause accumulation of dsRNA in infected cells. However, activation of the PKR-mediated antiviral response by dsRNA is counteracted by viral m142 and m143 proteins during MCMV infection. Both m142 and m143 proteins are important for MCMV replication and both of them are essential for inhibition of PKR and subsequent eIF2*α* phosphorylation [53]. To exert its role in inactivating PKR, m142 associates with m143 to form a stable heterotetramer complex including two molecules of m142, and each one binds to a monomer of m143. The m142-m143 multimer directly binds to PKR as well as to dsRNA to prevent PKR activation, resulting in relocalization of PKR into the nucleus and into an insoluble cytoplasmic compartment. Thus, PKR is unable to shut off cellular translation and to repress viral replication [54].

#### 2.2.4. ARV Nonstructural Protein p17

ARVs are double-strand RNA viruses that are classified as the members of Reoviridae family. ARV enters host cells via the caveolin 1-mediated endocytic pathway [55] and its replication occurs in the cytoplasm of infected cells [56]. The nonstructural protein p17 is encoded by the S1 genome segment of ARV with 146 amino acids [57]. It shuttles between the nucleus and the cytoplasm to affect signaling pathways involved in cell growth, paving a way for effective viral production [58, 59]. Our group has demonstrated that p17 is the protein responsible for ARV-induced host cell cycle arrest and autophagosome formation [22, 59]. Ectopically expression of p17 in both immortalized chicken embryo fibroblast (DF-1) cells and African green monkey kidney (Vero) cells by transfection with a p17-expressing plasmid caused phosphorylation of eIF2*α* that led to induction of autophagy [22]. It was also demonstrated that p17 triggered autophagy via activations of PTEN and AMPK to negatively regulate the function of mTORC1 [22]. We therefore considered that the p17 protein serves as an autophagy inducer in the life cycle of ARV.

### 2.3. Regulation of Autophagy Pathway

#### 2.3.1. The cFLIP-Like Viral Protein

Some *γ*-herpesvirus encode a viral protein homologous to the cellular FLICE-like inhibitor protein (cFLIP), such as KSHV vFLIP, herpesvirus saimiri (HVS) vFLIP, and molluscum contagiosum virus (MCV) 159L [60]. The cFLIP protein that has been identified exists in 3 forms based on their variable C-terminus. It also contains 2 death effector domains (DED) at the N-terminus. The FLIP is involved in negative regulation of cellular apoptosis induced by the activation of specific proapoptotic receptors on the cell surface, including Fas/CD95, DR4, and DR5 death receptors that associate with TNF-*α*-related apoptosis-inducing ligand (TRAIL), and tumor necrosis factor-*α* receptor 1 (TNFR1). Moreover, these ligand-receptor complexes interact with tumor necrosis factor receptor type 1-associated death domain protein (TRADD) and Fas-associated protein with death domain (FADD) to form the death-inducing signaling complex (DISC) which in turn activates the downstream caspase signaling cascade to induce apoptosis [61]. Due to structural similarity of FLIP to procaspase-8, which is as the initiator caspase in the caspase-dependent pathways, FLIP forms a heterodimer with procaspase-8, which becomes inactive, and therefore limiting the amount of procaspase-8 binding to DISC [62].

A recent study has unveiled another role of vFLIP in autophagy regulation. vFLIP can block cellular autophagy by binding Atg3 E2-like enzyme with its DEDs. Atg3 is a critical enzyme that mediates lipidation and conjugation of LC3 to promote autophagosome biogenesis. Interaction between vFLIP and Atg3 prevents LC3 from binding with Atg3. As a result, a reduction of autophagy occurs in virus-infected cells [60]. Exemplified by KSHV, a model was consequently proposed that it expresses vFLIP within viral latency program to subvert autophagy and to facilitate its own replication in virus-infected cells. Despite the ability of vFLIP binding to Atg3 to inhibit autophagy, Ritthipichai et al. have recently reported that rhesus monkey rhadinovirus vFLIP can enhance the autophagosome formation via an unknown mechanism in order to inhibit cellular apoptosis [63]. The functions of vFLIP expressed by different viruses seem somehow manifold in regulation of cell proliferation.

#### 2.3.2. CHIKV nsP2 Protein

CHIKV is an enveloped virus with a positive-strand RNA genome belonging to the* Alphavirus* genus. It triggers an autophagy response in infected human-cultured cells to promote viral replication [64]. CHIKV encodes 4 nonstructural proteins (nsP1 to nsP4) which are important for virus replication. During infection, nsPs bind to viral RNA to form replicative complexes (RC), thereby resulting in RCs to anchor into the subcellular membranes situated around the nucleus for viral protein synthesis. Among these nsPs, the nsP2 had been identified as an essential component of RCs [65]. Furthermore, the nsP2 can use the autophagy machinery to help virus replication through the binding to the human autophagy receptor NDP52. The experimental results of yeast two-hybrid system have shown that the C-terminal domain of nsP2 is responsible for the interaction with the coiled-coil domain of NDP52. In addition, it has been demonstrated that NDP52 colocalized with the* trans*-Golgi network associated RCs containing nsPs and dsRNA, while knockdown of NDP52 by using siRNA specific to NDP52 strikingly decreased CHIKV replication [66]. These data described above provide evidences suggesting that NDP52 can promote CHIKV replication via nsP2, although the detailed mechanism remains unclear.

#### 2.3.3. Viral Proteins That Interfere with the Progress of Autophagy

Many viruses have evolved to trigger an incomplete autophagy response in virus-infected cells to benefit their replications. For example, the M2 protein encoded by IAV is sufficient to induce cellular autophagy response [67]. Nevertheless, it can also block the fusion between autophagosomes and lysosomes, leading to accumulation of autophagosomes. Study of M2 protein has provided a possible mechanism in which blockade of autophagolysosome formation is through the interaction of M2 protein to Beclin 1 [68, 69]. Such blockade may enhance the retention time of viral proteins and RNA in infected cells. Rotavirus nsP4 protein can induce autophagy as well, and it binds to autophagosomes to prevent the autophagolysosome formation. The nsP4 protein of rotavirus is likely to recruit autophagosomal membrane to viroplasm to promote virus replication, wherein the viral RNA is replicated [70, 71]. Similarly, HIV-1 induces the autophagic vacuoles in macrophages during infection to enhance its replication, and its Nef protein blocks the maturation of autophagolysosomes to prevent Gag proteins from degradation [18, 72].

### 2.4. mTOR Signaling Pathway

Simian virus 40 (SV40) is a DNA virus that belongs to Polyomaviridae family. SV40, like other polyomaviruses, is potentially oncogenic (tumor causing). The small T antigen (ST) has 174 amino acid residues encoded by the SV40 early region. Studies that focused on the function of ST have linked the relationship between ST and the mechanism by which cancer cells can survive under condition of nutrient deprivation. ST binds to protein phosphatase 2A (PP2A), a primary serine/threonine phosphatase in mammalian cells, with its C-terminal domain to form a stable complex, disabling PP2A and resulting in disturbance of cellular progress [73]. Under glucose deprivation, the phosphorylation level of AMPK was increased in human foreskin fibroblasts expressing ST, and the rate of cell death was decreased in contrast to that of cells without expressing ST [74]. When AMPK is phosphorylated at Thr172 to become an active form, it directly phosphorylates TSC2 at Sre1387 and the mTORC1 subunit raptor at Ser722 and Ser792 to inhibit cell growth, thus inducing autophagy pathways for energy acquirement [75]. SV40 ST acts in cancer cells to maintain energy homeostasis during glucose deprivation by inhibiting the mTOR signaling pathway which is likely responsible for activation of autophagy.

### 2.5. Regulation of Cytoprotective Signaling Pathways

#### 2.5.1. The Unfolded Protein Response (UPR)

UPR is an alarm system for cells in response to the accumulation of unfolded or misfolded proteins in the lumen of the endoplasmic reticulum (ER), triggering formation of autophagy to digest and clean these unwanted proteins. There are three main pathways activated upon UPR induction, including transcription factor 6 (ATF6), PKR-like ER kinase (PERK), and inositol-requiring kinase 1 (IRE-1) [76]. A number of viruses that have been characterized encode proteins modifying autophagy through UPR to their own advantages during infection. For example, severe acute respiratory syndrome coronavirus (SARS-CoV) modulates UPR by activating PERK protein kinase by its viral spike (S) protein [77, 78], while the E protein encoded by the envelope gene downregulates the IRE-1 signaling pathway [79]. HCMV protein pUL38, like the small surface protein (HBs) of HBV, is capable of activating the key components of PERK pathway, including PERK, eIF2*α*, and transcription factor 4 (ATF4) [80, 81]. The latent membrane protein 1 (LMP-1) of EBV upregulates all three signaling pathways of UPR that have been described previously [76, 82]. Moreover, West Nile virus requires ATF6 signaling for its replication, and a viral protein responsible for regulating ATF6 signaling still remains to be determined [83]. On the contrary, HSV-1 glycoprotein B (gB), HCV cytosolic envelope protein E1, and CHIKV nonstructural protein 4 (nsP4) suppress the PERK pathway during infections [76, 84, 85].

#### 2.5.2. The DNA Damage Response (DDR)

DDR, a cellular self-protective mechanism, is responsible for detecting damaged DNA. Activation of DDR causes cell cycle arrest and initiates the DNA repair system, avoiding mutated DNA duplication. DDR signaling is primarily controlled by ataxia telangiectasia mutated (ATM), ATM and RAD3-related (ATR), and DNA-dependent protein kinase (DNA-PK) [87]. The existence of a complex interrelationship between viral infection and cellular DDR revealed that some viruses can selectively activate and/or repress DDR signaling pathways in a temporally coordinated manner to promote virus replication [87–91]. KSHV v-cyclin is one of the well-known viral proteins that trigger autophagy indirectly via activation of DDR. As the name implied, v-cyclin is homologous to D-type cyclins that binds to cyclin-dependent kinase 6 (CDK6) to form an active holoenzyme, allowing v-cyclin to constitutively deregulate cell cycle and to promote oncogenic stress. This stress triggers the DDR signaling, resulting in autophagy and cellular senescence [81, 86]. KSHV v-cyclin is cotranscribed with the latency-associated nuclear antigen (LANA) and vFLIP in a latent transcription unit, and it cooperates with vFLIP to precisely control the autophagy formation in KSHV-infected cells. This cooperation might contribute to the phenomenon observed in KSHV latent infection wherein the infected cells only have modest levels of autophagy and fail to senesce, as reported by Leidal et al. [92].

## 3. Conclusion

Due to the ability to degrade the pathogens from outer environment, autophagy is also one of the protective mechanisms against viruses that are harmful to the host. More and more studies revealed that viruses have evolved diverse mechanisms in order to evade from the defenses as autophagy. Nevertheless, previous investigations also revealed that several viruses induce autophagy to benefit themselves. The regulation of autophagy is a procedure involving a series of steps that require well-controlled signaling pathways. How the viruses operate in this process in order to complete their productive infection in host cells remain to be addressed. Understanding of the roles of autophagy and autophagy-related regulation in virus life cycle can raise the possibility in developing more specific antiviral treatments. And even more, the new findings might further support the therapies for other autophagy-related diseases, such as neurodegenerative disorders and cancer.

## Figures and Tables

**Table 1:** Role of the viral proteins in regulation of autophagy.

Viral protein	Virus	Activator or suppressor of autophagy	References
DP71L	ASFV	S^a^	[45]
p17	ARV	A^b^	[22]
nsP2	CHIKV	A	[66]
nsP4	CHIKV	S	[85]
LMP-1	EBV	A	[82]
HBs	HBV	A	[81]
HBx	HBV	A	[38]
TRS1	HCMV	S	[36]
PUL38	HCMV	A	[80]
E1 protein	HCV	S	[84]
Nef	HIV-1	A	[18, 72]
Glycoprotein B	HSV-1	S	[76]
ICP34.5	HSV-1	S	[43]
Us11	HSV-1	S	[48, 49, 51, 52]
vFLIP	HVS/KSHV/MCV	S	[60]
M2 protein	IAV	A	[67]
v-cyclin	KSHV	A	[81, 86]
vBcl-2	KSHV/*γ*HV-68	S	[9]
m142 & m143	MCMV	S	[53, 54]
IE180	PRV	S	[44]
nsP4	Rotavirus	A	[70, 71]
S protein	SARS-CoV	A	[77, 78]
Small T antigen	SV40	A	[73, 74]

^a^S: suppressor; ^b^A: activator.
